# COVID-19- related work, managerial factors and exhaustion among general practitioners in Sweden: a cross-sectional study

**DOI:** 10.1186/s12875-023-02228-w

**Published:** 2023-12-13

**Authors:** Helena Månsson Sandberg, Bodil J. Landstad, Åsa Tjulin, Emma Brulin

**Affiliations:** 1 Department of Health Sciences, Mid Sweden University, Östersund, Sweden; 2 Unit of Occupational Medicine, Karolinska Institutet, Stockholm, Sweden; 3 Faculty of Human Sciences, Mid Sweden University, Östersund, Sweden; 4 Unit of Research, Education and Development, Östersund Hospital, Östersund, Sweden

**Keywords:** COVID-19, Exhaustion, General practitioners, Management, Mental health, Occupational conditions, Sweden

## Abstract

**Introduction:**

A significant number of international studies show that general practitioners (GPs) suffered from burnout when working during the COVID-19 pandemic. A Swedish study found that more than 16% of GPs had exhaustion in spring 2021. Exhaustion can be regarded as an initial stage of burnout. A knowledge gap remains on GPs´ working conditions, the impact of management during the pandemic and how it was associated with exhaustion. This study aims to explore the association between severe symptoms of exhaustion and COVID-19 pandemic-related work and managerial factors among Swedish GPs and whether managerial factors have an impact on the association between exhaustion and COVID-19-related work factors.

**Methods:**

Cross-sectional data was drawn from the Longitudinal Occupational Health survey in Health Care Sweden (LOHHCS), which included a representative sample of practicing doctors in Sweden. The sample consisted of 6699 doctors with a response rate of 41.2%. This study constitutes a sample of doctors who reported working in primary care facilities at the time of data collection, i.e. 1013 GPs. The Burnout Assessment Tool (BAT) was used to assess severe symptoms of exhaustion. Questions were also asked about pandemic-related work and managerial factors. The data was analysed using descriptive statistics and multivariate logistic regression to identify the association between exhaustion, work and managerial factors.

**Results:**

The multivariate analysis showed that GPs who managed COVID-19 patients were about twice as likely to report severe symptoms of exhaustion. Further, GPs who reported that management was unsupportive, provided unsatisfactory working conditions and unsatisfactory policies for patient prioritisation were between two and four times more likely to report severe symptoms of exhaustion.

**Conclusions:**

COVID-19-related work and managerial factors had a significant impact on the mental health of GPs. Furthermore, the potentially protective effect that satisfactory management actions had on mental health was limited. In the aftermath of the COVID-19 pandemic and in preparation for future major crises that have a high impact on healthcare, there is a need to investigate the measures that can be taken to enable GPs to carry out their work, while maintaining their wellbeing.

## Introduction

In 2020, countries around the world were hit by the COVID-19 pandemic and this study will explore the impact working as a general practitioner (GP) during the pandemic had on their mental health. It is previously known that poor mental health is common among doctors. For instance, a systematic review including 182 articles from 45 different countries reveals a high prevalence of poor mental health, such as burnout, among all types of doctors [1]. The systematic review concludes that there is a variation in burnout across countries and that it is important to study contextual differences [1]. Until recently no study has explored the prevalence of exhaustion and clinical burnout and its potential antecedents among Swedish doctors, including GPs [2].

GPs, i.e., doctors working in primary care facilities, had a crucial role in limiting the impact of the COVID-19 pandemic at a community level [3]. For instance, in the primary care setting they were responsible for identifying risk groups and identifying new cases of COVID-19 while continuing to provide care to the general public [4]. Only a limited number of individuals with COVID-19 were hospitalised, instead, GPs managed the greatest share of the care related to COVID-19 [5, 6]. A significant number of international studies show that GPs suffered from burnout when working during the COVID-19 pandemic [7–17]. For this reason, the impact of the COVID-19 pandemic on burnout in GPs warrants further research to find out which work factors contributed to GP burnout, and whether managerial factors had an effect. This study will therefore focus on pandemic-related work factors, managerial factors and symptoms of exhaustion in Swedish GPs during the pandemic. In this study, exhaustion is regarded as a preliminary stage of clinical burnout [18].

### The concept of burnout and exhaustion

Schaufeli et al. [19] define clinical burnout as “… a work-related state of exhaustion that occurs among employees, which is characterised by extreme tiredness, reduced ability to regulate cognitive and emotional processes and mental distancing”. Thus, the inability to perform, as indicated by exhaustion and cognitive and emotional impairment, together with the unwillingness to perform, as indicated by mental distancing, constitute the four core symptoms of clinical burnout [19]. Schaufeli and Taris [20] argue that burnout is preceded by symptom development over time initiated by exposure to stressors, such as the high workload during the pandemic, and exhaustion. Exhaustion is defined as a “… severe loss of energy that results in feelings of both physical (tiredness, feeling weak) and mental (feeling drained and worn-out) exhaustion” [19]. Specific symptoms of exhaustion include a lack of energy to initiate work, feeling used up after a day of working, getting tired quickly even after minimal effort at work as well as the inability to relax after work [19]. Thus, exhaustion can be regarded as a preliminary stage of clinical burnout [18] and clinical burnout may implicate an evolution from exhaustion to cognitive and emotional impairment to mental distance, and back. However, the progression of these symptoms over time warrants longitudinal research [18].

A recent Swedish study explored all four symptoms of burnout risk, and found exhaustion among 13% of doctors in general, and among more than 16% of GPs in spring 2021 [2]. In contrast, there was a lower prevalence of the other symptoms of burnout (mental distance 8.2%, emotional impairment 1.7%, cognitive impairment 7.5%) among the GPs [2]. The prevalence of exhaustion among Swedish GPs was higher than in the general population before the pandemic [21]. Previous research has found that during the pandemic, the prevalence of poor mental health was higher among female GPs [22] and among GPs with fewer years of experience [23].

### Work factors of importance for healthcare professionals during the pandemic

During the COVID-19 pandemic, healthcare professionals in general struggled with excess workload [24]. GPs also reported higher workload during the pandemic than before the pandemic [25]. Studies show that the excess workload for GPs during the pandemic increased the risk of burnout [26, 27].

When working during these special circumstances, feelings of moral distress and emotional demands were common [24, 25, 28–31]. In addition, moral distress and emotional demands contributed to burnout [28, 29, 31–33]. Healthcare professionals felt moral distress when they could not provide the desired patient care due to lack of resources [34]. They felt alone in making critical decisions about which patients should be given access to medical resources [24, 35]. Additionally, GPs who managed COVID-19 patients reported more frequent symptoms of burnout and exhaustion, compared with those who did not personally manage these patients [13].

### The impact of management on healthcare professionals’ mental health during the pandemic

It has been well established that management-level actions are important to promote and maintain health among healthcare professionals [36–40]. For instance, burnout among doctors can be prevented if healthcare managers ensure they have a manageable workload [24, 38]. Support from their managers helps doctors to cope with work stress and to recover after stressful events, which also protects them against burnout [37]. This was also the case during the COVID-19 pandemic in terms of the impact of management measures on healthcare professionals’ mental health. A lower level of social support from management regarding the extent of the measures taken to protect the health of healthcare professionals was associated with mental health problems during the pandemic [41]. On the other hand, more supportive management, for instance through encouragement and provision of sufficient resources, helped to reduce work-related stress among healthcare professionals who worked during the COVID-19 pandemic [42]. Similarly, Feingold et al. [43] showed that feeling valued and supported by management was associated with a lower risk of mental health problems during the pandemic. In fact, management support was more important for healthcare professionals´ mental health than being married/having a partner and feeling adequately trained to perform required duties [43]. The importance of management support as a potentially protective factor for mental health problems was also emphasised by Zhang et al. [44]. In their study, support from management included access to appropriate personal protective equipment, access to up-to- date information and adequate communication [44].

Clinical management, including COVID-19 guidelines, was important to the mental health of healthcare professionals during the COVID-19 pandemic [8, 10, 24, 45]. GPs were uncertain about which COVID-19 guidelines they should adhere to [46]. This uncertainty was also associated with mental health problems among GPs [8, 10]. Hence, healthcare professionals appreciated when management provided them with frequent and relevant information related to the pandemic [45]. In fact, when managers were clear in decision-making, and included healthcare professionals in the process, mental health problems were prevented [24, 47].

Hospital-based doctors in Sweden reported shortcomings in management during the COVID-19 pandemic [35, 48]. They experienced that managers were not physically present and did not support their health and safety. Doctors were frustrated by not being heard by healthcare management and felt that their concerns and fears were ignored [35]. In addition, they experienced that management failed to provide necessary information and action plans [48].

### Linking work factors and managerial factors

As mentioned above, research has shown that both work and managerial factors impacted the mental health of healthcare professionals. However, management may also have affected work factors during the COVID-19 pandemic. For instance, previous research has confirmed the association between non-supportive management and moral distress among healthcare professionals [24, 28]. In contrast, effective and supportive management can decrease the moral distress of healthcare professionals [29, 32, 45]. Further, when managers provided clear guidelines for prioritisation in COVID-19- related care, the levels of moral distress also decreased [45].

### Primary care in Sweden

Sweden is divided into 21 self-governing regional authorities called regions. The regions are responsible for providing a significant proportion of all public healthcare services in hospitals and primary care facilities [49]. Primary care in the Swedish regions is the first-line care provided, covering medical treatment, preventive care and rehabilitation without any limitation in terms of illness, age or patient group [50]. Thus, the general purpose of Swedish primary care is to provide care for members of the general public that do not require hospitalisation [51]. Primary care collaborates with other levels of care and other authorities when needed in order to coordinate the care and treatment of patients [50]. The duties of GPs entail receiving patients for assessments, being on-call, participating in infection prevention efforts and being involved in crisis and disaster preparedness within the region [50].

However, it is not only the regions that are responsible for healthcare provision in the Swedish healthcare system, they share the responsibility with the local municipalities. Municipalities are responsible for elderly citizens with long-term care needs, such as those who live in nursing homes and those who receive home care support. Nevertheless, the regions are responsible for providing the municipality with medical resources [52], so most GPs serve citizens within the municipality-based elderly care [53]. Over the past decade, deficiencies have been highlighted in the cooperation between Swedish primary care and the municipal care of the elderly [54]. Due to a lack of time and resources, GPs do not have sufficient availability for elderly care provided through municipal services [54]. This was confirmed during the COVID-19 pandemic when major deficiencies were identified in the organisation of the Swedish municipality-based care of the elderly [55]. The prevalence of COVID-19 was high, as was the excess mortality in elderly care [56, 57]. It has been suggested many of the nursing home residents did not receive an individual assessment by a GP, and that relatives of palliative care residents were not involved in the end-of-life care [55]. Furthermore, there has also been debate about the fact that GPs had to make quick decisions, prioritise and carry out actions without sufficient evidence [58]. GPs in Sweden therefore requested increased support and clarity from the authorities [58].

In summary, previous international studies show that work factors, such as excess workload, moral distress and emotional demands, increased the risk of burnout among healthcare professionals that worked during the COVID-19 pandemic [26–29, 31–33]. Furthermore, healthcare professionals who reported that they experienced deficiencies in management during the pandemic, were also more likely to have symptoms of mental health problems [8, 10, 41]. In contrast, appropriate management actions were emphasised as potentially having a protective effect on the mental health of healthcare professionals [24, 42–44, 47]. However, most of these studies generally focus on healthcare professionals in the hospital setting. Therefore, a knowledge gap still remains about the working conditions and health of GPs during the COVID-19 pandemic [2]. This study aims to explore the association between severe symptoms of exhaustion and COVID-19 pandemic-related work and managerial factors among Swedish GPs and whether managerial factors have an impact on the association between exhaustion and COVID-19- related work factors.

## Methods

### Sample and procedure

This study focuses on GPs working in Sweden, i.e., all doctors who reported working in a primary care facility at the time of data collection. Data was drawn from the Longitudinal Occupational Health survey in Healthcare Sweden (LOHHCS) study, which included a representative sample of practicing doctors in Sweden [2]. The sample in the LOHHCS study was drawn from the Swedish Occupational Register, with a stratified random sampling method based on a total of 12 strata (six times two). For geographical stratification, the population was stratified based on six administrative healthcare regions. Furthermore, with respect to place of work, two strata were applied: either primary care facilities or hospitals. Based on these 12 strata and a 50% response rate, a power calculation suggested a sample of 7200 doctors.

Between February and May of 2021, 7200 doctors received an invitation to participate in the LOHHCS study, of which 501 did not match the inclusion criteria and were removed from the sample. Thus, the sample consisted of 6699 doctors with a response rate of 41.2%. This study constitutes a sample of doctors who reported working in primary care facilities at the time of data collection, i.e., 1013 GPs.

## Measurements

### Outcome variable

The first dimension of the Burnout Assessment Tool (BAT) was used to assess exhaustion [19]. The BAT comprises 23 items divided into four core dimensions, one of which is exhaustion measured by 8 items (e.g., “At work, I feel mentally exhausted”, “Everything I do at work requires a great deal of effort”, “After a day at work, I find it hard to recover my energy”).

The other BAT dimensions are mental distance (5 items), emotional impairment (5 items) and cognitive impairment (5 items). Each item is rated on a five-point scale from 1 (never) to 5 (always). Exhaustion showed high internal consistency (Cronbach’s *a* = 0.927). A total mean value was obtained based on the eight items. The BAT construct has been psychometrically validated in other countries [18, 59] and is currently being validated in Sweden.

Following Schaufeli et al. and de Beer et al. [18, 59], exhaustion was dichotomized with a cut-off value set at 3.31. Values above or equal to 3.31 indicated severe symptoms of exhaustion (1). Values below 3.31 indicated no or mild symptoms of exhaustion (0).

### Explanatory variables

#### Pandemic-related work factors

Respondents were asked about whether they managed COVID-19 patients (i.e., have you managed patients who have been diagnosed with COVID-19 or are waiting for test results?). The question was answered on a 5-point scale; 1 being “yes during the whole pandemic” and 5 being “no”. The variable was dichotomised into 0 = occasional work shifts or no, and 1 = yes, during the whole pandemic, yes, but not anymore or yes, right now but not at the beginning.

Prior to the LOHHCS data collection, interviews were carried out with 40 doctors with varying specialisms, about their work-related experiences of the first wave of the pandemic [35, 48]. Based on these interviews, questions were developed concerning work during the pandemic. This study used these COVID-19- related questions concerning work and managerial factors.

*Moral distress* was measured using two questions. The respondent was asked to rate how stressful they experienced it was to (1) deny relatives visits to seriously ill in-patients due to COVID-19 restrictions, and (2) deny patients with COVID-19 more advanced care due to a lack of resources. They responded on a scale from 1 “not stressful” to 4 “very stressful”. A fifth alternative was “not relevant” which was coded as “not stressful” in this study. No studies exist on the clinical cut-off of moral distress. The total mean of these two questions was therefore computed and based on the distribution of mean values, the cut-off score was set to 1.99 (last quartile), with values above the cut-off score being reported as stressful (1).

To explore *emotional demand*, respondents were asked to rate whether their work was more or less emotionally demanding due to the pandemic (i.e., has your work been more or less emotionally demanding during the COVID-19 pandemic than before?). They responded on a 5-point scale, 1 being “much more demanding” and 5 being “much less demanding”. The variable was dichotomised into 0 = unchanged, less demanding or much less demanding, and 1 = more demanding or much more demanding. To explore *excess workload* respondents were asked to rate whether their perceived amount of work increased or decreased due to the pandemic (i.e., has the COVID-19 pandemic resulted in your working more or less?). They responded on a 5-point scale ranging from 1 being “much more” to 5 “much less”. Answers reporting a decrease in perceived amount of work were removed from the sample (n = 100; 9.0%) as the reason for the GPs to report reduced perceived amount of work was unknown. For instance, it may have been due to poor health rather than the pandemic. The variable was dichotomised into 0 = unchanged and 1 = more or much more.

#### Pandemic-related managerial factors

In the interviews carried out with doctors prior to the LOHHCS data collection, it appeared that management was important in relation to doctors´ work during the COVID-19 pandemic. Some of these experiences have been further explored in this study [35, 48]. Three managerial factors were explored; supportive management, work environment management and clinical management. Each of these factors was explored using two variables. First, *supportive management* was measured using one question asking the GPs whether their immediate manager was physically present in the clinic (i.e., my immediate manager is physically present during the COVID-19 pandemic) and one question asking whether their manager was supportive (i.e., my immediate manager supports me during the COVID-19 pandemic). Both questions were answered on a 4-point scale ranging from often to never. These two variables were each dichotomised into 0 = often or sometimes, and 1 = rarely or never. *Work environment management* was measured using two questions asking GPs to rate how they experienced management conduct in relation to utilisation of staff resources and working conditions (i.e., how do you perceive the management’s skills during the COVID-19 pandemic regarding use of staff resources and working conditions for staff?). These two questions were answered on a 5-point scale, 1 being “very good” and 5 being “very poor”. The variables were dichotomised into 0 = very good, good and neutral, and 1 = fairly poor and very poor. Finally, *clinical management* was measured using two questions focusing on the existence of policies for patient prioritisation and treatment guidelines (i.e., how do you perceive the management´s skills during the COVID-19 pandemic regarding policies for patient prioritisation and treatment guidelines?). These two variables were also answered on a 5-point scale: 1 being “very good” and 5 “very poor”, and dichotomised into 0 = very good, good and neutral, and 1 = fairly poor and very poor.

#### Covariates

*Gender* was reported as male and female and the participants were also asked about their *hierarchical position*. The variable was dichotomised with trainee doctors coded as 0 = junior doctors, and specialists and consultants as 1 = senior doctors. In Sweden, doctors have about seven years of training before they have a certificate of specialisation as general practitioners. Once they have finished specialist training, they become specialists.

### Statistical analysis

Descriptive statistics were used to describe the characteristics of the sample and to identify the prevalence of severe symptoms of exhaustion across exposure variables and covariate variables, reported through frequencies (n) and percentages (%).

A variance inflation factor (VIF) and matrix correlation were applied to assess multi-collinearity. The highest correlation coefficient was 0.620, and a VIF of between 1.015 and 1.799 indicated that multi-collinearity was not present.

Logistic regressions were conducted to identify associations between exhaustion and work and managerial factors. First, univariate logistic regressions were used to explore the association between exhaustion and each of the explanatory and covariate variables. Secondly, a multivariate logistic regression was used to assess the effect of each variable on exhaustion, adjusted for the effects of the other independent variables.

The multivariate logistic regression was carried out in six different models. The reason for performing the analyses in separate models is that it makes it possible to control for different categories of variables at different steps. In Model 1, the variables *managing COVID-19 patients*, *gender* and *hierarchical position* were entered. Work factors in terms of *moral distress*, *emotional demands* and *excess workload* were added to these in Model 2. In order to test each of the managerial factors in relation to exhaustion, we added them separately in Models 3 to 5. In Model 3, *supportive management* (physically present manager and supportive manager) was added, in Model 4, *work environment management* (use of staff resources and working conditions) was tested, and in Model 5 we tested *clinical management* (policies for patient prioritisation and treatment guidelines). Finally, in Model 6 all variables were tested simultaneously.

The logistic regression was reported with odds ratios (OR) and 95% confidence intervals (CI). The level of significance was set to *p* = .05. The overall model fit was assessed by using 2-log likelihood (-2LL) and Nagelkerke R-Square. To remove the effect of the stratified sampling method, all analyses were adjusted for the six strata reflecting administrative healthcare regions. All statistical analysis was performed using the Statistical Package for Social Sciences, SPSS (version 27.0) for Mac.

## Results

Table 1 shows that the prevalence of severe symptoms exhaustion among GPs working in Sweden in spring 2021 was 14.4%. The prevalence was 16.4% among female GPs and 11.6% among male GPs. Furthermore, 17.0% of junior GPs had severe symptoms of exhaustion, and among their senior colleagues the prevalence was 13.8%.

**Table 1:** Demographic and pandemic-related work and management characteristics of severe symptoms of exhaustion among Swedish general practitioners (n = 1013)

Variable	N	All	Exhaustion
**Total**	1013		14.4%
*Gender*			
Female	590	58.2%	16.4%
Male	423	41.8%	11.6%
*Hierarchical position*			
Junior doctor	313	31.7%	17.0%
Senior doctor	675	68.3%	13.8%
**PANDEMIC- RELATED WORK FACTORS**			
*Managing COVID-19 patients*			
Rarely/No	254	25.1%	7.8%
Yes	756	74.9%	16.7%
*Moral distress*			
Not stressful	684	68.1%	11.9%
Stressful	320	31.9%	20.1%
*Emotional demands*			
Unchanged/decreased	661	65.4%	12.0%
Increased	349	34.6%	18.8%
*Excess workload*			
Unchanged	655	64.7%	11.5%
Increased	358	35.3%	19.9%
**PANDEMIC- RELATED MANAGEMENT FACTORS**			
**Supportive management**			
*Physically present manager*			
Yes	893	91.1%	13.5%
No	87	8.9%	26.7%
*Supportive manager*			
Yes	815	86.2%	12.8%
No	130	13.8%	29.1%
**Work environment management**			
*Use staff resources*			
Neutral/good	854	87.3%	12.1%
Fairly/very poor	124	12.7%	31.7%
*Working conditions*			
Neutral/good	748	75.9%	8.9%
Fairly/very poor	237	24.1%	33.0%
**Clinical management**			
*Policies on patient prioritisation*			
Neutral/good	755	77.8%	10.8%
Fairly/very poor	216	22.2%	29.2%
*Treatment guidelines*			
Neutral/good	786	81.0%	11.9%
Fairly/very poor	184	19.0%	26.7%

In terms of pandemic-related work factors, the prevalence of severe symptoms exhaustion was higher among GPs who managed COVID-19 patients (16.7%) than among GPs who did not (7.8%). Further, among those GPs who felt moral distress, 20.1% also had severe symptoms of exhaustion, while for those who stated that they did not feel moral distress the prevalence of symptoms was 11.9%. Of those who experienced increased emotional demands during the pandemic, 18.8% had severe symptoms of exhaustion, while the prevalence was 12.0% of those who stated unchanged or less emotional demands. Furthermore, 19.9% of GPs who reported excess workload due to the pandemic also had severe symptoms of exhaustion, and amongst those who stated that their workload was unchanged, the prevalence was 11.5%.

One thing that was common to all pandemic-related managerial factors was that across all variables, GPs who reported a greater dissatisfaction with management also reported a higher prevalence of severe symptoms of exhaustion. The prevalence of severe symptoms exhaustion among GPs who experienced a lack of management support was 29.1%, while for those who stated that they had received good support the prevalence was 12.8%. 33% of GPs who stated that management action was unsatisfactory when it came to working conditions also reported severe symptoms of exhaustion, compared to 8.9% of those who responded that it was satisfactory. Among GPs who experienced that management provided unsatisfactory policies on patient prioritisation, 29.2% had severe symptoms of exhaustion versus 10.8% of those who stated that such policies were satisfactory. Moreover, the prevalence of severe symptoms exhaustion among GPs who experienced that management provided unsatisfactory treatment guidelines was 26.7%, while for those who responded that management provided satisfactory guidelines, the prevalence was 11.9%.

### Association between severe symptoms of exhaustion and pandemic-related work and managerial factors

All values of the independent variables, except hierarchical position, in the univariate logistic regression showed a significant association with severe symptoms exhaustion (Table 2). Results are consistent with the descriptive statistics and show that COVID-19 pandemic-related work and managerial factors were associated with severe symptoms of exhaustion among GPs during the pandemic. Thereafter, the multivariate logistic regression was computed (Table 2). Adjusting for gender and hierarchical position, Model 1 shows that GPs who managed COVID-19 patients were more than twice as likely to report severe symptoms of exhaustion compared to those who did not manage COVID-19 patients (OR = 2.32, 95% CI: 1.39–3.90). Further, being a female GP was associated with higher odds of severe symptoms exhaustion (OR = 1.54, 95% CI: 1.05–2.26).

**Table 2:** Multivariate logistic regression for the associations between severe symptoms of exhaustion and COVID-19 pandemic-related work and management factors (n = 1013)

Variable	Univariate	Model 1	Model 2	Model 3	Model 4	Model 5	Model 6
Total (n)		n = 964	n = 956	n = 890	n = 928	n = 908	n = 844
**PANDEMIC-RELATED WORK FACTORS**							
Managing COVID-19 patients (ref. not managing COVID-19 patients)	2.31 (1.38–3.85)	2.32 (1.39–3.90)	1.99 (1.18–3.38)	2.21 (1.26–3.88)	1.79 (1.03–3.09)	2.04 (1.16–3.50)	2.11 (1.15–3.88)
Moral distress (ref. no distress)	1.85 (1.28–2.68)		1.56 (1.06–2.29)	1.49 (0.99–2.23)	1.37 (0.91–2.06)	1.53 (1.02–2.29)	1.34 (0.87–2.05)
Emotional demands (ref. unchanged/decreased)	1.76 (1.22–2.55)		1.35 (0.90–2.02)	1.27 (0.83–1.94)	1.15 (0.75–1.77)	1.35 (0.89–2.05)	1.23 (0.78–1.92)
Excess workload (ref. unchanged workload)	1.94 (1.35–2.78)		1.35 (0.90–2.02)	1.48 (0.98–2.23)	1.48 (0.98–2.25)	1.47 (0.98–2.21)	1.37 (0.89–2.12)
**PANDEMIC-RELATED MANAGEMENT FACTORS**							
*Supportive management*							
No present manager (ref. present manager)	2.26 (1.32–3.84)			1.50 (0.80–2.33)			1.05 (0.53–2.07)
No support from manager (ref. had support)	2.84 (1.81–4.43)			2.30 (1.37–3.86)			1.22 (0.68–2.18)
*Work environment management*							
Unsatisfactory use of staff resources (ref. satisfactory)	3.30 (2.12–5.14)				1.44 (0.84–2.44)		1.14 (0.62–2.06)
Unsatisfactory working conditions (ref. satisfactory)	5.33 (3.63–7.82)				4.01 (2.54–6.33)		3.37 (2.00-5.67)
*Clinical management*							
Unsatisfactory policies on patient prioritisation (ref. satisfactory)	3.40 (2.32–4.99)					2.90 (1.74–4.83)	1.95 (1.12–3.39)
Unsatisfactory treatment guidelines (ref. satisfactory)	2.68 (1.79–4.01)					1.18 (0.69–2.03)	0.84 (0.47–1.52)
**COVARIATES**							
Female (ref. male)	1.52 (1.04–2.23)	1.54 (1.05–2.26)	1.52 (1.03–2.25)	1.57 (1.04–2.36)	1.36 (0.90–2.05)	1.47 (0.98–2.20)	1.40 (0.91–2.15)
Senior doctor (ref. junior)	0.86 (0.57–1.28)	0.92 (0.62–1.38)	0.90 (0.59–1.35)	0.99(0.65–1.52)	1.15 (0.74–1.78)	0.98 (0.64–1.51)	1.23 (0.77–1.95)
Model fit Nagelkerke R-Square 2-log likelihood		0.052 780.667	0.083 756.883	0.112 704.855	0.174 686.675	0.140 701.211	0.195 627.897

In Model 2, the results show that, the OR for GPs who managed COVID-19 patients decreased in relation to Model 1 (OR = 1.99, 95% CI: 1.18–3.38), however, with overlapping CI. In addition, in Model 2, moral distress remains significant (OR = 1.56, 95% CI: 1.06–2.29), while emotional demands and excess workload became non-significant, compared with the univariate analyses.

Pandemic-related management factors were then explored in Models 3 to 6. After adjusting for supportive management (Model 3), the results showed that GPs who managed COVID-19 patients were 2.21 times more likely to report severe symptoms of exhaustion and that the OR slightly increased in relation to the OR in Model 2. Meanwhile, the other pandemic-related work factors (moral distress, emotional demands, and excess workload) became non-significant. Emotional demands, and excess workload remained non-significant in Models 4 to 6. These work factors thus do not have any significant impact on severe symptoms of exhaustion when adjusting for the other variables. Further, GPs who experienced their manager as being unsupportive (Model 3) were more than twice as likely to report severe symptoms of exhaustion (OR = 2.30, 95% CI: 1.37–3.86). The experience of a physically absent manager becomes non-significant.

When adjusting for work environment management in Model 4, the likelihood of GPs who managed COVID-19 patients reporting severe symptoms of exhaustion decreased in relation to Model 2, to 1.79 times more likely, but the CI largely overlaps. Model 4 shows that GPs who reported that management action was unsatisfactory when it came to working conditions were four times more likely to report severe symptoms of exhaustion (OR = 4.01, 95% CI: 2.54–6.33). How management utilized staff resources becomes non-significant.

After adjusting for clinical management in Model 5, the results showed that GPs managing COVID-19 patients were two times more likely to report severe symptoms of exhaustion (OR = 2.04, 95% CI: 1.16–3.50). Furthermore, moral distress had a significant association to severe symptoms of exhaustion (OR = 1.53, 95% CI: 1.02–2.29). The results show that GPs who stated that management had unsatisfactory policies for patient prioritisation were almost three times more likely to report severe symptoms of exhaustion (OR = 2.90, 95% CI: 1.74–4.83). Unsatisfactory treatment guidelines became non-significant.

In the final Model 6, all COVID-19 pandemic-related work and managerial factors were added. OR for managing COVID-19 patients were basically the same as in Model 2 indicating that pandemic-related management factors had little impact on the association between managing COVID-19 patients and severe symptoms of exhaustion. Moral distress, emotional demands and excess workload was non-significant. Furthermore, adjusting for all management factors, Model 6 shows that no support from manager became non-significant while the other management factors remained non-significant or significant. GPs who reported that management provided unsatisfactory working conditions (OR = 3.37, 95% CI: 2.00- 5.67) and unsatisfactory policies for patient prioritisation (OR = 1.95, 95% CI: 1.12–3.39) were significantly more likely to report severe symptoms of exhaustion.

## Discussion

In this study we explored the associations between severe symptoms of exhaustion and COVID-19 pandemic-related work and managerial factors among Swedish GPs. The results showed that among the pandemic-related work factors managing COVID-19 patients and to some extent moral distress had an association with severe symptoms of exhaustion. In specific, GPs who managed COVID-19 patients had approximately twice the odds of reporting severe symptoms of exhaustion. Also, unsatisfactory management (i.e., supportive, work environment and clinical management) increased the likelihood of reporting severe symptoms of exhaustion. Furthermore, the study aim was also to explore if managerial factors had an impact on the association between exhaustion and COVID-19- related work factors. Results indicate that management factors did not impact this association.

The increased likelihood of reporting exhaustion among GPs who managed COVID-19 patients is consistent with studies from other countries [7–17]. Furthermore, in line with one previous study [13], GPs who managed COVID-19 patients were more likely to report exhaustion compared to GPs who did not treat COVID-19 patients. However, as the assessment of burnout differs between studies it is difficult to compare the results [1]. For instance, in Rotenstein et al.’s systematic review the prevalence of burnout among doctors ranged from 0 to 80.5% and the prevalence of exhaustion ranged from 0 to 86.2%. In 85.7% of the studies included, the Maslach Burnout Inventory was used (MBI) [1]. The MBI does not have clinical cut-off values for exhaustion and burnout, which may have an impact on reported prevalence [1].

Exhaustion is one dimension of burnout and can be regarded as a preliminary stage of burnout [18]. Having severe symptoms of exhaustion thus means an increased risk of developing burnout later [18]. Therefore, the results of this study indicating that 16.7% of GPs who managed COVID-19 patients experienced severe symptoms of exhaustion raises concerns, because even before the onset of the COVID-19 pandemic, it was known that many Swedish GPs were at risk of exhaustion, burnout and sick leave [60]. Longitudinal studies are thus needed to investigate the symptom development in GPs over time. Also, this study only covers the first year of the pandemic raising concerns of potential effect on burnout risk after continuing working with COVID-19patients.

In other words, does the extreme work situation during the pandemic that is associated with exhaustion prevail and cause symptom development and increase the risk of future burnout?

The significant association between managing COVID-19 patients and severe symptoms of exhaustion among the GPs in our study may partly be explained by the role GPs have in municipality-based elderly care. In Sweden during the COVID-19 pandemic, the situation within elderly care was ethically demanding for GPs, entailing difficult medical decisions regarding the frail and elderly [58]. For instance, Swedish GPs responsible for care in nursing homes have been accused of denying COVID-19 patients hospital-based care, and even guilty of euthanasia [58]. Further, there has been criticism about the fact that relatives of palliative-care residents were not involved in the end-of-life care [55]. Not being able to give patients the care needed may cause moral distress among the GPs [58]. Future studies should further explore how GPs experienced working in elderly care and how that impacted their health.

Previous studies show that supportive management led to a decreased risk of mental health problems among healthcare professionals working during the COVID-19 pandemic [42–44]. Also, studies show that burnout among doctors can be prevented if management facilitates a reduced workload with sufficient time for recovery [24, 38]. However, the absence of a potential protective effect of supportive management on exhaustion, shown in our results, indicates that supportive management is only one factor that can help employees through an extreme workload. Thus, different types of management support, or a supportive work environment in general is not enough for those dealing with an extreme work situation such as the COVID-19 pandemic. For instance, one longitudinal study shows that a high level of work demand is associated with a higher risk of burnout, regardless of the level of support in the work environment [61]. In other words, despite the existence of trusting collaboration in the work group and the possibility to decide how work should be carried out, as long as work demand remains high, employees still have a higher risk of burnout [61]. Similar results have been demonstrated in other longitudinal studies [62, 63]. It may therefore be assumed that the GPs who managed COVID-19 patients were under substantial pressure in their daily work during the COVID-19 pandemic. In addition, GPs had insufficient time for their own recovery [10]. Overall, our study indicates that support from management was not sufficient to protect GPs who managed COVID-19 patients from exhaustion even though it has previously been demonstrated that healthcare management must be accessible and visible during pandemics [24].

Having had unsatisfying working conditions had a direct negative association with severe symptoms of exhaustion among the studied GPs. The great importance of satisfactory working conditions during the pandemic is a recurring theme among Swedish hospital doctors [35, 48]. In fact, the absence of an occupational health and safety mindset from management can jeopardise the health of doctors [48].

This study also shows that GPs who experienced unsatisfactory clinical management, i.e., policies on patient prioritisation were more likely to suffer from exhaustion, as indicated in previous studies [8, 10]. It is therefore critically important that difficult decisions, such as patient prioritisation, are made in organised fashion and clear guidelines are essential [45, 64]. It has been shown that clear COVID-19 guidelines for patient prioritisation decreased moral distress among Norwegian doctors [45]. However, our result showed that clinical management did not have any potentially protective effect on the negative effects from managing COVID-19 patients nor to moral distress. Furthermore, previous research show that it was important to include healthcare professionals in management decisions during the COVID-19 pandemic as this also decreased the risk for burnout [47]. A previous Swedish study revealed that GPs were dissatisfied with the clinical management during the COVID-19 pandemic and disagreed somewhat with the top-down instructions [46]. A similar situation was described among hospital doctors in Sweden [35, 48]. This implies that further research would be valuable to investigate if, and in what ways, GPs can be involved in decision-making processes and preparation for major future crises.

In summary, the overall experience of Swedish doctors was that healthcare services were not prepared for a major crisis such as the COVID-19 pandemic [35, 46, 48], and management at all levels faced extensive challenges [48]. The question to be asked is whether these extraordinary circumstances made it even more difficult for management to provide GPs with healthy and sustainable working conditions. Future studies should further explore if, and in what ways, management can minimise the risk of poor mental health for GPs during a major crisis that has a high impact on healthcare services.

### Strengths and limitations

This cross-sectional study illustrates work and managerial factors in relation to exhaustion among Swedish GPs from February to May 2021, which limits the usability of the findings in terms of causal inference.

There are no validated instruments that measure working conditions during the specific conditions that prevailed within healthcare during the COVID-19 pandemic. None-validated explanatory variables were therefore used, which might be a limitation of this study. However, the explanatory variables used are considered highly relevant as they were based on a large number of interviews with Swedish doctors of varying specialties, carried out in the early phase of the pandemic.

To exclude those GPs who reported a decrease in amount of work might be a limitation, as it excluded 9% of the sample. However, it was considered more correct to exclude them as the reason for the GPs to report reduced perceived amount of work was unknown, and nothing we investigated further.

The dichotomisation of the variables might be another limitation of the study as it leads to some loss of information when compared to a continuous measure of exhaustion. However, dichotomous definitions of burnout can be considered as more practical when identifying doctors with burnout, or as in our case, severe symptoms of exhaustion and at risk of future burnout [1]. Dichotomisation of the exhaustion variable based on the cut-off score was thus considered appropriate. The explanatory variables were also considered appropriate for dichotomisation as they were of a more categorical character indicating more or less of the particular factor.

The clinical cut-off scores of the BAT have not yet been assessed on the Swedish population, and in our study, we therefore used the cut-off scores of BAT based on the Dutch population [18, 59]. However, the cross-national measurement invariance of the BAT has been successfully demonstrated across seven different countries (i.e., Belgium, The Netherlands, Germany, Austria, Finland, Ireland and Japan), and it is thus possible to use the BAT instrument to assess and compare burnout levels across countries [59]. Furthermore, until clinical cut-off values exist in Sweden, this study indicates that we can apply cut-off values from other European countries. It should be mentioned that an association between exhaustion and depression has been found among healthcare professionals [65]. When interpreting the results, the potential overlap between these two conditions has to be taken into consideration.

One of the strengths of the study is the representative sample that reflects the population of GPs in Sweden. Further, a slightly higher response rate than 41.2% would have been desirable. Nevertheless, given that the survey was conducted during the ongoing pandemic, the response rate is considered to be satisfactory.

## Conclusions

This study showed that working with COVID-19 patients and pandemic-related managerial factors had a significant association with the mental health of GPs during the pandemic. Furthermore, the potentially protective effect that satisfactory management actions may have on mental health was limited.

In this study the BAT instrument was used to measure exhaustion among GPs. Due to different measures of burnout used in research, it is difficult to compare the prevalence of exhaustion among the GPs in our study with other studies on GPs. This indicates that more research is needed about exhaustion and its potential antecedent factors among GPs, and with advantage use the BAT.

Further, future studies should explore how exhaustion among GPs unfolds over time, after the pandemic. For instance, do the severe symptoms of exhaustion GPs had in the acute phase of the pandemic turn into a clinical burnout with severe consequences? The associations between work and managerial factors related to the pandemic and exhaustion is a cause for concern, and it may have a negative impact on the quality of care. In the aftermath of the COVID-19 pandemic and in preparation for future major crises that have a high impact on healthcare, there is a need to investigate the measures that can be taken to enable GPs to carry out their work, while maintaining their wellbeing.

## Data Availability

The datasets generated and analysed during the current study are not publicly available. Respondents have consented to the publication of aggregated data, but not to open publication of data for individuals. Data is available from the corresponding author on reasonable request.

## Abbreviations

BAT: Burnout Assessment Tool
CI: Confidence interval
GP: General practitioner
LOHHCS: Longitudinal Occupational Health survey in Healthcare Sweden
OR: Odds ratio
VIF: Variance inflation factor
